# Ultra-Low Power Wearable Infant Sleep Position Sensor

**DOI:** 10.3390/s20010061

**Published:** 2019-12-20

**Authors:** Inyeol Yun, Jinpyeo Jeung, Mijung Kim, Young-Seok Kim, Yoonyoung Chung

**Affiliations:** 1 Department of Electrical Engineering, Pohang University of Science and Technology, Pohang, Gyeongbuk 37673, Korea; inyul1225@postech.ac.kr (I.Y.); jpjeung@postech.ac.kr (J.J.); 2 Korea Sport Industry Development Institute, Pohang University of Science and Technology, Pohang, Gyeongbuk 37673, Korea; mijungkim@postech.ac.kr (M.K.); andyyskim@postech.ac.kr (Y.-S.K.)

**Keywords:** power switch, antenna radiation with polymer encapsulation, infant sleep position sensor

## Abstract

Numerous wearable sensors have been developed for a variety of needs in medical/healthcare/wellness/sports applications, but there are still doubts about their usefulness due to uncomfortable fit or frequent battery charging. Because the size or capacity of battery is the major factor affecting the convenience of wearable sensors, power consumption must be reduced. We developed a method that can significantly reduce the power consumption by introducing a signal repeater and a special switch that provides power only when needed. Antenna radiation characteristics are an important factor in wireless wearable sensors, but soft material encapsulation for comfortable fit results in poor wireless performance. We improved the antenna radiation characteristics by a local encapsulation patterning. In particular, ultra-low power operation enables the use of paper battery to achieve a very thin and flexible form factor. Also, we verified the human body safety through specific absorption rate simulations. With these methods, we demonstrated a wearable infant sleep position sensor. Infants are unable to call for help in unsafe situations, and it is not easy for caregivers to observe them all the time. Our wearable sensor detects infants’ sleep positions in real time and automatically alerts the caregivers when needed.

## 1. Introduction

Recently, a variety of wearable sensors have been developed and attracted much attention in medical/healthcare/wellness/sports applications [1,2,3]. Wearable sensors conveniently provide major physical information, such as electrocardiogram (ECG), electromyogram (EMG) [4], electroencephalogram (EEG) [5], body fat [6], and blood glucose level [7] in real time without bulky instruments. In addition, most wearable sensors support a wireless communication function to transfer the measured data for further analysis and processing [8]. As prevention, rather than treatment, has become emphasized, wearable sensors are increasingly being utilized in modern medicine [9]. However, most previous wearable sensors have critical drawbacks in terms of user convenience because of significant power consumption with various functionalities, large battery volume, rigid/bulky form factors, and frequent charging cycles [10,11,12]. In previous studies, people sustained efforts to develop self-powered wearable sensors using piezoelectric/triboelectric materials and solar cells [13,14,15]. However, they did not generate enough power to operate the sensors, and thus an additional power source was required. In addition, several studies used a low-power simple chip to reduce the power, but this approach still required large-capacity battery and frequent charging cycle [16,17]. In other studies, wireless power transmission was attempted, but the available range and the efficiency were still too low to be used in practical use [18,19]. In addition, the radiation efficiency of the antenna was not carefully considered in previous wireless wearable sensors [20]. In a previous study, an antenna pattern was designed and deployed on flexible substrate for wireless data transmission, but the researchers did not analyze the negative effects of sensor encapsulation on the radiation performance [21]. As radiation characteristics are an important factor for low power consumption, as well as for high-performance wireless communication, the degradation caused by the encapsulation materials must be overcome.

In this study, we developed a method for reducing the power consumption and improving the antenna radiation characteristics for a wearable sensor and utilized these technologies to monitor infant sleep position. It is generally well known that infants are safe when sleeping on their backs, as their breathing can be disturbed by soft objects in a prone position, which can even lead to death. This case is a typical example of sudden infant death syndrome (SIDS), and 40 out of 100,000 infants died of SIDS in the United States in 2016 [22]. In order to prevent SIDS, it is essential to maintain a supine sleeping position rather than a prone position. In the United States, infant mortality has reduced by 50% through the “Safe to Sleep” campaign, which encourages parents to have their infants sleep on their backs [22]. Despite of this campaign, many infants still die from the wrong sleeping position. Several approaches have been proposed for infant sleep monitoring. First, a sleep position is monitored through a camera, and a prone position can be detected by image processing. However, this method requires bulky instruments in a fixed place [23]. Another approach is to use a sleep position detector based on an accelerometer. As infants are sensitive to the touch, a comfortable fit is important. Although the accelerometer-based position detecting method is simple in terms of checking the infant’s sleep position, the position detectors in previous studies had bulky form factors and short battery life [24,25,26]. Therefore, it is necessary to develop a reliable infant sleep monitoring system with outstanding usability. In this paper, we present a wearable sensor that can easily monitor the infant sleep position, solving the conventional problems associated with power consumption and the antenna radiation characteristics. In order to reduce the power consumption of the wearable sensor, we introduced a base station as a signal repeater, which is connected to an external power source. We also devised a special switch that supplies electricity to high-power components only when they need to be on. Because our wearable sensor exhibited extremely low power consumption, the total power could be supplied by a low-capacity flexible paper battery. In addition, we observed that the antenna radiation characteristic was degraded by a polymeric encapsulation layer that is commonly used for a comfortable fit. Our encapsulation patterning method improved the antenna radiation while maintaining good wearability. Finally, the effects of radiation from the wireless sensor on the human body were studied through radio frequency (RF) simulations. 

## 2. Materials and Methods

### 2.1. Fabrication of Wearable Sleep Monitoring Sensor

**Figure 1:**
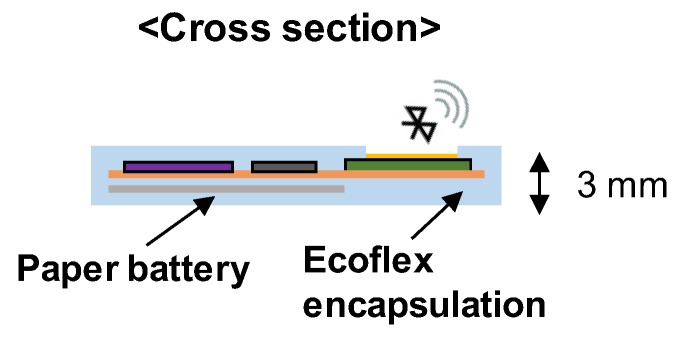
Schematic of the wearable sleep monitoring sensor.

The wearable sleep monitoring sensor consisted of a flexible printed circuit board (FPCB) assembled with chips, a paper battery, and polymer encapsulation (see Figure 1). First, a commercial accelerometer sensor (Analog Devices, ADXL335, Norwood, MA, USA), inverter (NXP Semiconductors, 74HC14, Eindhoven, The Netherlands), relay (MEDER electronics, CRR03-1A, Cincinnati, OH, USA), microcontroller unit (ATMEL, ATmega328P-PU, San Jose, CA, USA), Bluetooth RF module (Waveshare, NRF24L01, Shenzhen, China), and a paper battery were soldered on a FPCB. A mixture of silicone (Ecoflex, 00-30, Smooth-On, Macungie, PA, USA) was poured into a rectangular parallelepiped mold. After removing the bubbles in a vacuum desiccator, the Ecoflex and FPCB were combined and cured for 4 h. For the local patterning in encapsulation, an antenna-shaped aluminum mold was placed onto the antenna, and the Ecoflex was poured, followed by curing for 4 h. The length and width of the wearable sensor was 5 and 3 cm, respectively, and the thickness was approximately 3 mm.

### 2.2. Measurement of Dielectric Constant and Loss Tangent

PDMS and Ecoflex were prepared in a toroidal form with 10 mm thickness. After calibrating a network analyzer (8720D, Keysight, Santa Rosa, CA, USA) with calibration kit (HP 85050C, Keysight, Santa Rosa, CA, USA), each specimen was inserted into a waveguide-shaped line, and the 2-port S-parameter was measured. The complex relative permittivity and relative permeability were calculated using the measured S-parameters.

### 2.3. Power Consumption Measurement

The power consumption of each chip was measured by a semiconductor analyzer (B1500A, Keysight, Santa Rosa, CA, USA). The semiconductor analyzer supplied a certain voltage required by each chip, and the current flowing into the chip was measured. Then, the power consumed by each chip was calculated by multiplying the voltage and the current.

## 3. Results

### 3.1. Low-Power Operation System

In portable devices, power consumption is one of the most dominant factors that affects users’ convenience. Wearable sensors have more limitations because of their small size and flexibility preference. In most portable sensors with various functionalities or wireless capability, a battery occupies a significant portion of the total volume. We adopted a thin paper battery (23 mm × 48 mm × 0.45 mm) to improve the flexibility and to reduce the size significantly. As the form factor and the capacity of battery have trade-off relations, a low-power operation is essential in wearable devices. We measured the power consumption of each component to establish a strategy to reduce the total power of the sensor. During the measurements, the wearable sensor was set to be in a prone position, and an alarm message was sent through the RF module. As shown in Table 1, the microcontroller unit (MCU) and RF module consume a significant amount of power compared to the accelerometer.

**Figure 2:**
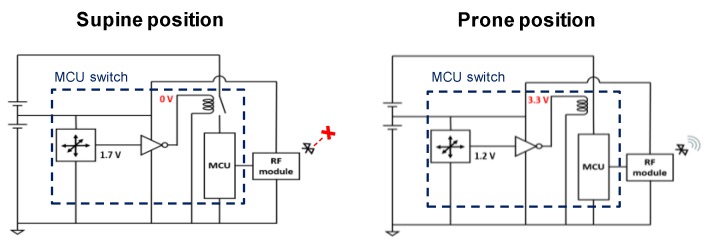
Overall circuit diagram of wearable sleep monitoring sensor. The low-power accelerometer detects infant’s sleep position, and the MCU switch supplies power to the MCU only when the infant is in a prone position.

We implemented an electrical switch that supplies power to the MCU and RF module only when the infant is in a prone position. In other words, the MCU switch, consisting of an inverter and a relay, allows power to the energy-intensive components only when they need to be on. A metal–oxide–semiconductor field-effect transistor (MOSFET) switch could be used instead of the relay; however, an additional power source is required to operate the transistor in saturation mode for reliable power supply. We simply configured the switch using a relay device without additional components. Figure 2 explains the operation of the MCU switch. The output of the accelerometer, which detects the infant’s sleep position, is 1.7 V when the infant sleeps on his/her back. It drops to 1.2 V when the infant is in a prone position. The inverter converts the accelerometer output into either 0 or 3.3 V. Then, the relay connects and disconnects the power supply to the MCU and the RF module, depending on the output of the inverter. 

**Figure 3:**
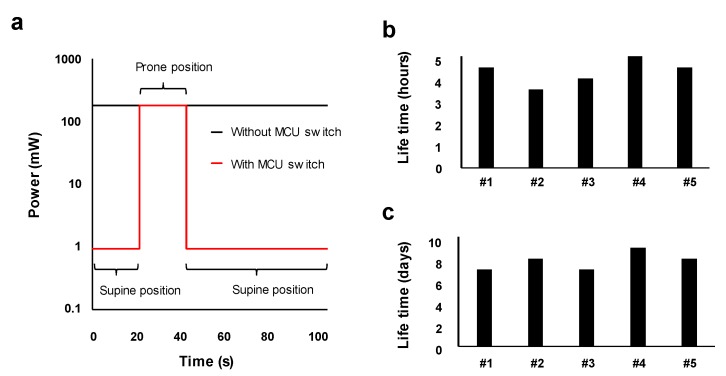
(**a**) Comparison of total power consumption with and without MCU switch. The MCU switch significantly reduces the power consumption in idle state. Measured lifetime of the paper battery utilized in the wearable sensor (**b**) without MCU switch and (**c**) with MCU switch. The average lifetime is only 4 h without the MCU switch, but it lasts over a week with the switch.

This MCU switch, which consumes less than 1 mW per one switching cycle, significantly reduces the total power consumption of the sensor. Without the MCU switch, the sensor consistently consumes 180 mW and operates for less than 5 h with the paper battery (see Figure 3a,b). However, with the MCU switch, the total power is reduced to being only 1 mW in an idle state when the wireless function is not needed. The incident of rolling over rarely occurs, but is fatal with infants [27]. To test the battery life time, we assumed an extreme condition that the infant’s body flips twice an hour. For babies under six months, an average sleep time is approximately 15 h a day [28]. We designed a battery test equipment that flipped the wearable sensors twice an hour and then observed an operating time without battery change. As shown in Figure 3a, the average power consumption of our device was found to be 0.94 mW. As the paper battery capacity was found to be 115.5 mWh, the theoretical battery life time was estimated to be 8.2 days. We experimentally confirmed that our sensor can be used for 7.8 days on average without charging, as shown in Figure 3c. We greatly reduced the power consumption of the wearable sensor by controlling the power supply to the energy-intensive components with simple circuitry. For this sleep monitoring application, the caregivers can receive an alarm message immediately through their smartphone by charging the wearable sensor only once a week.

### 3.2. Antenna Radiation Characteristics

Most commercial RF modules are designed and mass-produced, assuming that an antenna pattern is exposed to air. However, the radiation is often hindered by additional layers between the antenna and the propagation medium. We performed high-frequency simulations to study how RF signals from the antenna are attenuated by a polymer layer for encapsulation. In addition, we improved the antenna radiation characteristics by a local patterning approach.

The relative permittivity and loss tangent of Ecoflex and polydimethylsiloxane (PDMS) polymers were measured at several representative frequencies for wireless communication (see Table 2). The loss tangent is defined as the imaginary part of relative permittivity divided by the real part. The lower the loss tangent value, the better the encapsulation performance with less absorption loss. The loss tangent of Ecoflex was found to be 0.014, which shows a relatively poor propagation behavior compared to free space. 

**Figure 4:**
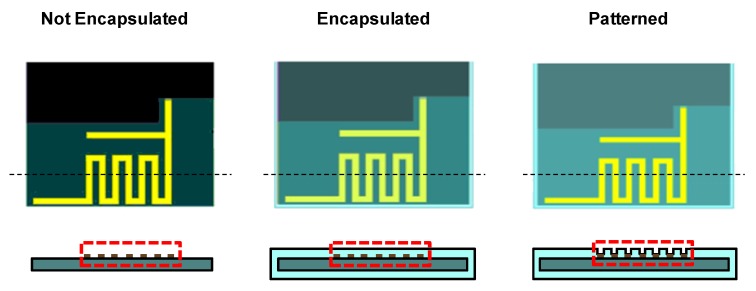
Simulation structures for antenna radiation analysis. When the structure is not encapsulated, the whole area is exposed to air. When the structure is encapsulated, the entire device is covered with polymeric material (Ecoflex). When the encapsulated structure is patterned, only the antenna pattern is exposed to air and the other area is covered with Ecoflex.

Using the ANSYS HFSS program, we calculated the antenna performance in three cases: no encapsulation, full encapsulation with polymer, and locally patterned encapsulation, as depicted in Figure 4. In the patterned sample, the encapsulating polymer right above the Bluetooth antenna was locally removed.

**Figure 5:**
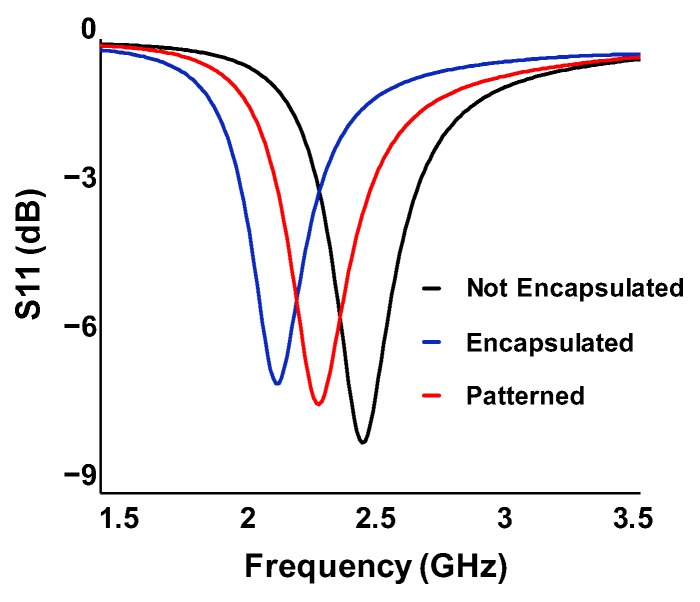
S11 parameter vs. frequency for each antenna structure. The value at the Bluetooth communication frequency (2.4 GHz) was calculated to be −8.16 dB (not encapsulated), −2.77 dB (encapsulated), and −6.04 dB (patterned).

The S-parameter describes the input–output relationship between each port in an electrical system, which is usually represented by the ratio of input to output power. The S11 parameter, in particular, represents how much power is reflected from a device under test, and thus provides the relative radiated power from the input. The smaller the S11 value, the better the radiation characteristics of antenna at a certain frequency. As shown in Figure 5, the S11 of the RF module without any encapsulation was −8.16 dB at 2.4 GHz. A full encapsulation of the Ecoflex degraded the S11 value to be −2.77 dB, which indicated that the radiation efficiency was deteriorated by more than half.

**Figure 6:**
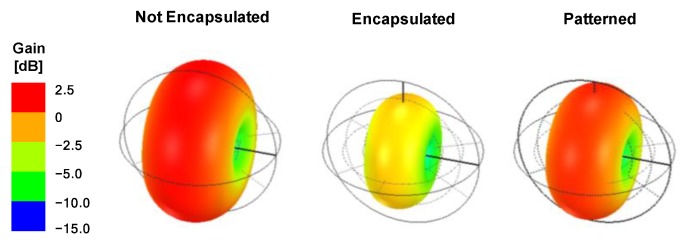
Antenna three-dimensional radiation pattern at 2.4 GHz for each structure. Maximum gain of each structure was 0.04 dB (not encapsulated), −3.22 dB (encapsulated), and −0.54 dB (patterned).

Although the RF performance was significantly decreased by the polymer encapsulation, it was essential to achieve a thin and soft form factor for the wearable device. To solve this problem, we locally patterned the encapsulation with the same lateral shape as the antenna. This approach provided a soft encapsulation while maintaining the intrinsic antenna characteristics. With the patterning, the S11 value was greatly enhanced to be −6.04 dB at 2.4 GHz. As shown in Figure 6, we calculated the antenna gain, defined as the product of beam directivity and radiation efficiency, for each structure. When the full encapsulation was applied, the maximum antenna gain was decreased by 3.26 dB due to the polymer component. However, the gain was decreased by only 0.58 dB using the local patterning method. In particular, this local patterning can be applied more efficiently to a broadside antenna whose radiation pattern was perpendicular to the substrate. The improvement of the radiation characteristics enables wireless communication with less energy; the output power of the RF module can be decreased by 40% with patterned encapsulation, compared to the module with full encapsulation, assuming the same radiation power. We confirmed that the degradation of RF performance due to encapsulation can be overcome by a suitable local patterning approach.

### 3.3. Specific Absorption Rate (SAR) Simulation

**Figure 7:**
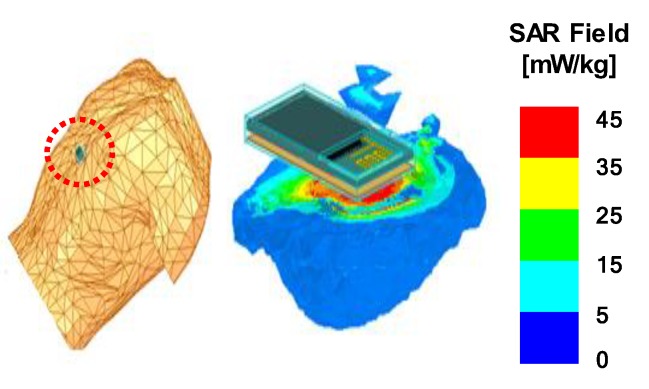
Specific absorption ratio (SAR) of the sensor on the human body model for each antenna structure. The excitation power was adjusted to have the same maximum gain of 0 dB. The maximum SAR value of each structure was 0.337 W/kg (not encapsulated), 0.471 W/kg (encapsulated), and 0.254 W/kg (patterned).

We calculated the specific absorption rate (SAR), which represents the amount of electromagnetic energy absorbed into the body [29]. The simulation was performed assuming that our sensor was located within 5 mm from the chest of the human torso model provided by the ANSYS HFSS [30]. As shown in Figure 7, the SAR maximum value of the non-encapsulated structure was calculated to be 0.337 W/kg, assuming that the output power of RF module was 1 mW. However, in the case of the encapsulated structure, more RF power was required to compensate the attenuation by the polymeric layer. As a result, the encapsulated structure required 2.12 times higher power to maintain the same radiation in the air, which increased the maximum SAR value to be 0.471 W/kg. However, as our locally patterned structure consumed only 1.14 times more power than the sample without encapsulation, its maximum SAR value was only 0.254 W/kg. The lowest SAR value in the locally patterned structure was attributed to the attenuation of electromagnetic waves towards the body. We noted that this RF power can provide a Bluetooth communication range of up to 55 m in an open outdoor environment [31]. The international commission on non-ionizing radiation protection (ICNIRP) has set a SAR limit of 2 W/kg, regardless of age and health [32]. The calculated SAR value is sufficiently low compared to this permissible standard value, which indicates that our wireless sensor is safe to be used near the skin [33].

## 4. Infant Sleep Monitoring System Demonstration

**Figure 8:**
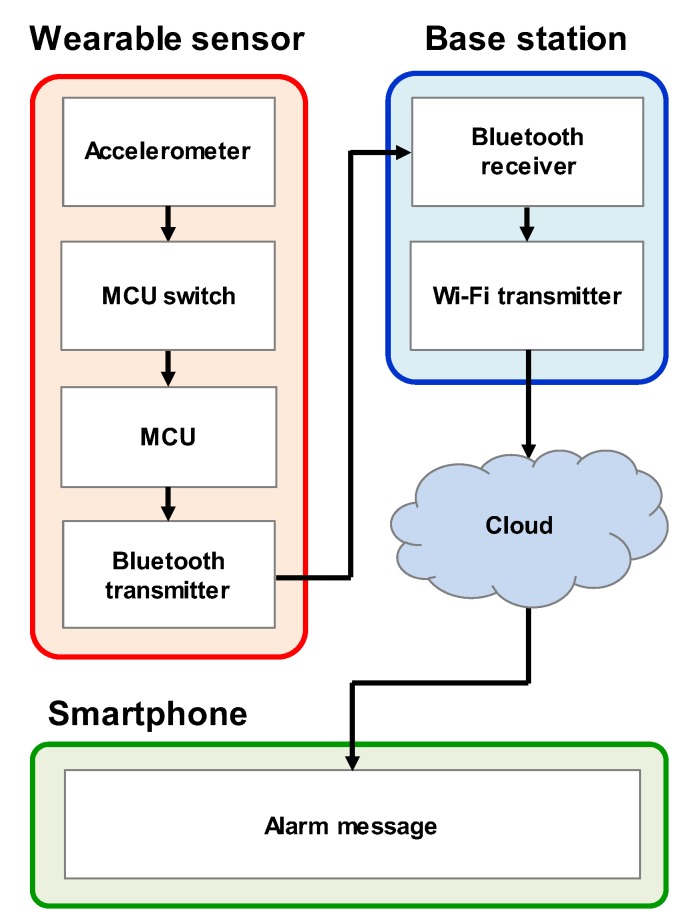
Block diagram of the infant sleep monitoring system. The wearable sensor detects a sleeping position and sends a RF signal to the base station when the infant sleeps on his/her stomach. The base station transmits the signal from the wearable sensor to the caregiver’s smartphone through Wi-Fi. Then, the smartphone rings so that the caregiver can immediately check the infant’s status.

The sleep position monitoring system was composed of a wearable sensor, base station, and smartphone, as shown in Figure 8. In the wearable sensor part, the infant’s sleep position is detected by an accelerometer sensor. An electrical switch supplies power to the MCU only when the infant is in a prone position. Because the MCU, which consumes the largest amount of power, is powered only when the wireless communication system needs to operate, the total power consumption is greatly reduced. When the MCU is on, it sends a wireless signal to the base station using a Bluetooth transmitter. The base station is a repeater that can be powered by an outlet or a high-capacity battery. It consists of a Bluetooth receiver, MCU, and Wi-Fi transmitter. When the base station receives a signal from the wearable sensor, it sends an alarm message to the caregiver’s smartphone through Wi-Fi communication. Then, the caregivers can come to the infant immediately and lay him/her on his/her back. The wearable sensor and caregiver’s smartphone could communicate directly without the base station, but this configuration significantly increases the power consumption of the wearable sensor. Because the base station takes full responsibility for the communication through the cloud, the wearable sensor can maintain a low-power state.

**Figure 9:**
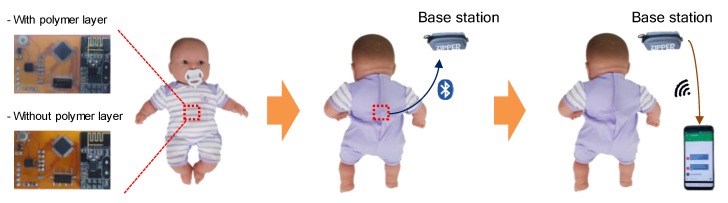
Demonstration of the infant sleep monitoring system. The infant doll was placed on its back. When the doll was rolled over, the sensor inside the clothes detected the inappropriate position and sent a signal to the base station through Bluetooth. The sensor was embedded in the red dashed box. The base station transmitted the signal from the sensor to the caregiver’s smartphone through Wi-Fi.

We demonstrated our wearable sleep monitoring system with an infant doll (see Figure 9). When the doll was inverted, the wearable sensor detected the position and sent a signal to the base station. When the base station received the signal, it transmitted a signal to a cloud server through Wi-Fi that the doll was in a prone position. The cloud server then sent an alarm message to the caregiver’s smartphone and let the caregiver immediately check the position. We tested the operation of the sensor in different situations. First, we rotated the wearable sensor at 3, 10, 20, and 30 rpm to check the effects of the rotational speed. The wearable sensor accurately detected the infant doll’s position regardless of the speed. This accuracy resulted from the fact that our sensor detected the position on the basis of the direction of gravity, not the speed or acceleration of the movement. For the same reason, if the infant doll was not in a prone position after turning over, the sensor did not send an alarm message. The latency between turning over and alarm warning on the smartphone was measured to be 2.6 s on average; this delay was dominated by Bluetooth pairing between the wearable sensor and the base station.

## 5. Discussion

In this work, we improved the practical usability of wearable sensors by achieving low power operation and high antenna radiation efficiency. We significantly reduced the power consumption with a special switch. In general wearable sensors, central processor and RF communication module consume the most electric energy. We devised a switch circuitry that powers the energy-intensive components only when they need to operate, thereby reducing the power to be less than 1 mW. With the low power requirement, thin and flexible materials were used as the power source, usually a bottleneck of wearable sensor’s form factor. Soft polymeric layers were used to enhance users’ comfortable fit in wearable applications. However, this encapsulation severely reduces the radiation efficiency for wireless communication. We improved the antenna radiation characteristics through a localized patterning approach, maintaining a soft encapsulation. Thus, the enhanced form factor can provide comfortable use of wearable sensors in daily life.

## Figures and Tables

**Table 1:** Measured average power consumption of major components in the wearable sensor.

	Power Consumption (mW)
Accelerometer	0.81
Microcontroller unit (MCU)	154.14
Radio frequency (RF) module	39.29

**Table 2:** Relative permittivity and loss tangent of Ecoflex.

Frequency (MHz)	Ecoflex	
	ε/ε_0_	tan δ
868 ^1^	2.814	0.018
900 ^2^	2.813	0.018
915 ^3^	2.813	0.018
2400 ^4^	2.811	0.014
5000 ^5^	2.809	0.016

Center frequency of ^1^ Zigbee in Europe, ^2^ Lora, ^3^ Zigebee in United States, ^4^ Bluetooth and Wi-Fi, and ^5^ Wi-Fi.
